# Multifaceted effects of synthetic TLR2 ligand and *Legionella pneumophilia *on Treg-mediated suppression of T cell activation

**DOI:** 10.1186/1471-2172-12-23

**Published:** 2011-03-24

**Authors:** Wendy WC van Maren, Stefan Nierkens, Liza W Toonen, Judith M Bolscher, Roger PM Sutmuller, Gosse J Adema

**Affiliations:** 1 Department of Tumor Immunology, Nijmegen Centre for Molecular Life Sciences, Radboud University Nijmegen Medical Centre, Nijmegen, the Netherlands; 2 Schering-Plough Research Institute, Target Discovery Oss, Molenstraat 110, 5340 BH Oss, The Netherlands

## Abstract

**Background:**

Regulatory T cells (Treg) play a crucial role in maintaining immune homeostasis and self-tolerance. The immune suppressive effects of Tregs should however be limited in case effective immunity is required against pathogens or cancer cells. We previously found that the Toll-like receptor 2 (TLR2) agonist, Pam3CysSK4, directly stimulated Tregs to expand and temporarily abrogate their suppressive capabilities. In this study, we evaluate the effect of Pam3CysSK4 and *Legionella pneumophila*, a natural TLR2 containing infectious agent, on effector T (Teff) cells and dendritic cells (DCs) individually and in co-cultures with Tregs.

**Results:**

TLR2 agonists can directly provide a co-stimulatory signal inducing enhanced proliferation and cytokine production of naive CD4+ Teff cells. With respect to cytokine production, DCs appear to be most sensitive to low amounts of TLR agonists. Using wild type and TLR2-deficient cells in Treg suppression assays, we accordingly show that all cells (e.g. Treg, Teff cells and DCs) contributed to overcome Treg-mediated suppression of Teff cell proliferation. Furthermore, while TLR2-stimulated Tregs readily lost their ability to suppress Teff cell proliferation, cytokine production by Teff cells was still suppressed. Similar results were obtained upon stimulation with TLR2 ligand containing bacteria, *Legionella pneumophila*.

**Conclusions:**

These findings indicate that both synthetic and natural TLR2 agonists affect DCs, Teff cells and Treg directly, resulting in multi-modal modulation of Treg-mediated suppression of Teff cells. Moreover, Treg-mediated suppression of Teff cell proliferation is functionally distinct from suppression of cytokine secretion.

## Background

The immune system is of crucial importance to our health and survival. Faced with pathogenic threats from outside as well as the rise of cancer cells from within, our immune defense must be able to cope with very diverse opponents. Mammals have developed a diverse set of receptors that sense components derived from pathogens and damaged cells. Amongst the best studied receptors are the so called pattern recognition receptors (PRR) like the Toll-like receptor (TLR) family, RIG-I-like receptor (RLR) family and the NOD-like receptor (NLR) family of proteins [1]. In general, engagement of these receptors on immune cells results in their activation, like enhanced antigen presentation, inflammatory cytokine production and the acquisition of immune effector function [2].

Pathogen recognition through specific TLRs can be of crucial importance for the induction of protective immunity. For instance, TLR4-deficient mice are more susceptible for infections with *Neisseria meningitidis*, *E. coli*, *Haemophilus influenzae*, *Salmonella enteritidis*, and *Klebsiella pneumonia *[3]. In this regard, the immunological effects of TLR2 ligation are remarkably different compared to the other TLRs (reviewed by Netea et al [3]). Firstly, TLR2 has been reported to direct the broadest repertoire of danger-associated molecular patterns from a large variety of pathogens, including gram-positive and gram-negative bacteria, fungi, viruses, and parasites, but also endogenous proteins like Heat Shock Protein 60 (HSP60) [4]. This broad range of recognition may be explained by the heterodimerization of TLR2 with either TLR1 or TLR6. However, the recent publication of the TLR1/2 receptor-ligand crystal structure [5], in combination with the extremely high affinity of TLR2 for its lipoprotein ligands [6], increases the possibility that a number of putative TLR2-ligands have no intrinsic TLR2-activating capacities but were actually contaminated by lipoproteins [6]. Secondly, TLR2-deficient mice are less susceptible to lethal infections with *Aspergillus fumigatus, Yersinia enterocolitica *or *Candida albicans*, which is in contrast with *e.g. *TLR4-deficient mice [7]. In TLR2-deficient mice, resistance to *C. albicans *is mediated by a stronger Th1 response due to diminished production of IL-10 during the infectious challenge [8]. The distinct roles of TLR2 and TLR4 in immunomodulation was further emphasized by findings that TLR2-deficient mice experienced increased joint inflammation in preclinical rheumatoid arthritis (RA) models, while TLR4-deficient mice were more resistant [9]. Interestingly, the enhanced immunological responses in TLR2-deficient mice correlate with decreased numbers of Tregs in these mice [8]. Moreover, *C. albicans *induced proliferation and survival of Tregs in a TLR2-dependent manner [8].

Different types of Tregs have been characterized and these Tregs are indispensable for the maintenance of immunologic self-tolerance and immune homeostasis [10]. The naturally occurring CD25+CD4+FoxP3+ Tregs are generated in the thymus and constitute about 5-15% of the peripheral CD4+ T cells in healthy animals and humans [11-13]. Once naturally occurring Tregs are activated via TCR-triggering, they are able to actively suppress the function of multiple immune cells, such as CD4+CD25-effector T cells (Teffs) and antigen presenting cells (APCs). Although these activities are essential for maintaining tolerance and preventing autoimmunity, their suppressive capacity may interfere with the development of a potential anti-tumor/anti-pathogen immune response, implicating the need for a mechanism that regulates Tregs. We recently demonstrated that TLR2 triggering on Tregs by Pam3Cys in combination with T-cell receptor (TCR) activation resulted in proliferation of the otherwise non-proliferating Tregs and, importantly, the temporal abrogation of their suppressive capabilities [14]. After a resting period, the Tregs regain their suppressive, non-proliferative phenotype, indicating this is a reversible process.

Since TLR2 is widely expressed in immune cells, we now investigated the consequences of TLR2-signaling on different immune subsets involved in adaptive immune responses; APCs, Teffs and Tregs. We found increased TLR2 expression on activated immune cells. Interestingly, although TLR2 triggering abrogated Treg-mediated suppression of Teff cell proliferation, TLR2 triggering was unable to restore effector cytokine secretion. In addition, we show that Pam3Cys acts on Tregs, Teffs and APCs to reverse the suppressive effects of Tregs. Similar results were found with the pathogen *Legionella pneumophila *(HKLP) containing potent TLR2 ligands [15-17], emphasizing the crucial role of TLR2 in immunomodulation in infections.

## Results

### TLR2 expression and effects on Treg, Teffs and DCs

**Figure 1:**
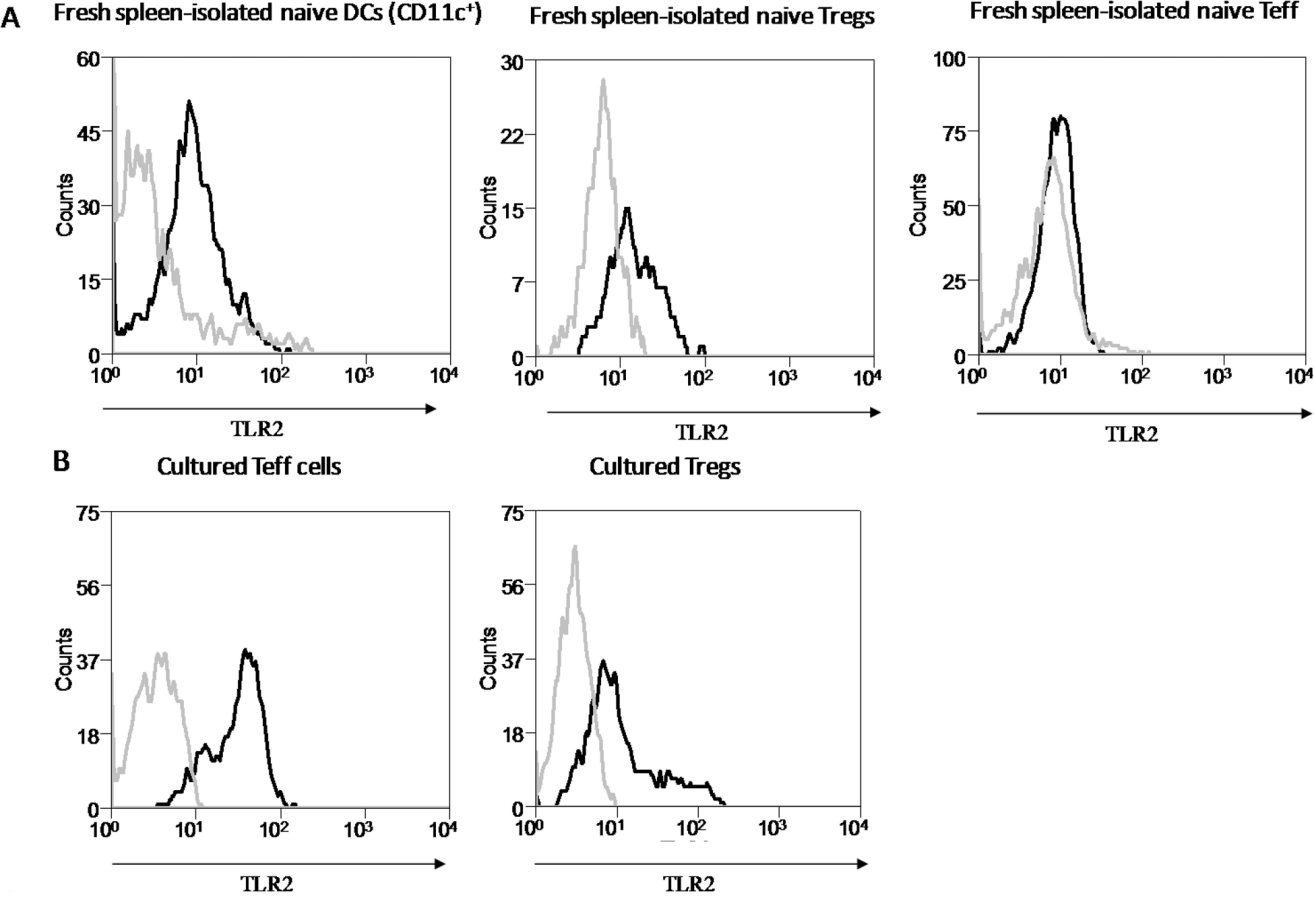
**TLR2 expression profile on fresh and cultured immune cells**. (A) Dendritic cells (CD11c^+^), CD4^+^CD25^+^Foxp3^+ ^Treg and CD4^+ ^effector T cells (Foxp3^-^) were isolated from spleens of naïve mice and immediately analyzed for TLR2 surface expression by flow cytometry (grey line, isotype control; black line, TLR2 antibody). TLR2 is expressed on freshly isolated DCs, while expression on Teff and Treg is absent or low. (B) Treg and Teff were cultured in the presence of Pam3Cys, anti-CD3 and irradiated splenocytes as APCs for several weeks (see materials and methods). Activation induced TLR2 expression on both Treg and Teff which remained high when cultured for several weeks. Representative results of 3 independent experiments are shown, all experiments were performed in duplicate.

To decipher the role of TLR2 in immune responses in more detail, the expression and function of TLR2 on CD4+CD25+Foxp3+ Tregs, CD4+ Teffs and DCs were studied. Expression of TLR2 is readily detected on the cell surface of freshly isolated DCs (Figure 1A), while expression on freshly isolated CD4+ Teffs and Tregs is low or absent (Figure 1A). In line with the data from Liu *et al. *[18], TLR2 expression is up-regulated upon activation of these T cells (data not shown), and both CD4+ T cell populations continue to express TLR2 when cultured for several weeks with CD3 and Pam3Cys (Figure 1B).

**Figure 2:**
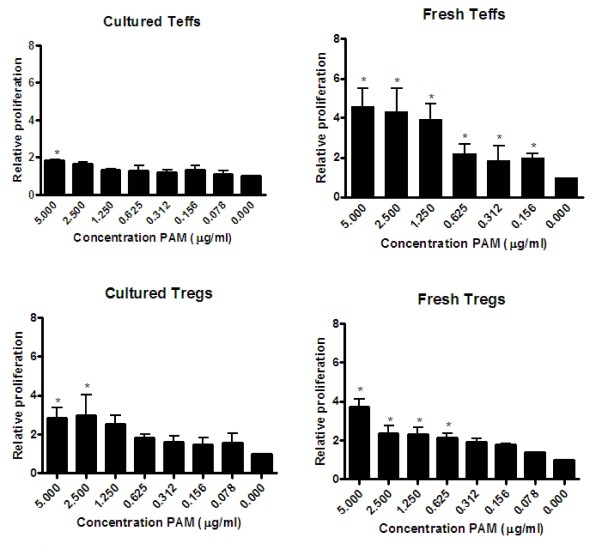
**Effect of Pam3Cys titration on proliferation of fresh versus cultured Tregs and Teff cells**. Teff and Treg were freshly isolated from spleen or cultured for weeks and stimulated with different concentrations of Pam3Cys in the presence of 1 μg/ml anti-CD3. After 4 days proliferation was determined using thymidine incorporation (relative expression compared to anti-CD3 alone). The condition without Pam3Cys is set to 1 and the conditions with Pam3Cys are related to this condition. The absolute cpm levels of the cells with (5 ug/ml) and without Pam3Cys are for cultured Teffs 13383 cpm vs 7456 cpm, for fresh Teffs 31076 cpm vs 6693 cpm, for fresh Treg 15590 cpm vs 3776 cpm, and cultured Treg 16642 cpm vs 4936 cpm. Freshly isolated Tregs and Teff cells showed increased proliferation upon TLR2 stimulation, while only cultured Tregs (not cultured Teff) remained sensitive to TLR2 triggering. Representative results of 3 independent experiments are shown. The error bars represent standard deviations of triplicate measurements.

Since the expression of TLR2 on these T cell subsets seems to be affected by their activation state, we investigated how TLR2 expression correlated to the functional responsiveness of DCs, Teffs and Tregs to the TLR2 ligand Pam3Cys. As shown in Figure 2, both freshly isolated Tregs and naive Teffs showed enhanced proliferation after CD3 stimulation in the presence of increasing amounts of TLR2-ligand, indicating Pam3Cys co-stimulated these T cells. In the absence of CD3 stimulation, no proliferation was observed on either Teffs or Tregs (data not shown), which is in line with the absence of TLR2 up-regulation on these cells. Analysis of the cultured CD4+ T cell subsets revealed that only the cultured Tregs, not the cultured Teff cells, remained responsive towards TLR2 stimulation. This finding is consistent with our current understanding that primed Teff cells become less dependent on co-stimulatory signals, as has been described for CD28 stimulation.

**Figure 3:**
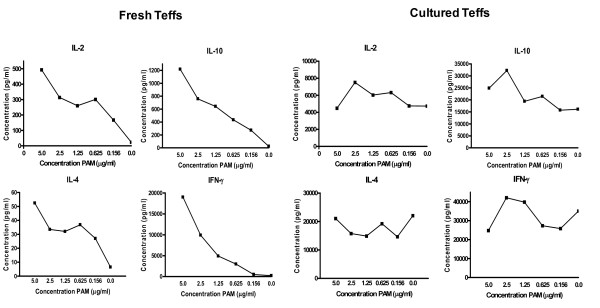
**Cytokine production of Teffs upon TLR2-triggering**. Freshly isolated and cultured Teff were stimulated with anti-CD3 and increasing concentrations of Pam3Cys. Fresh Teff were additionally cultured in the presence of irradiated APCs that did not produce these cytokines upon stimulation (data not shown). Freshly isolated Teff showed a clear dose dependent increase in IL-2, IL-4, IL-10 and IFNγ. Cultured Teff cells produced high amounts of cytokines in the absence of Pam3Cys and failed to show a clear effect of Pam3Cys. All cytokine profiles were determined by Luminex analyses. Representative results of 2 independent experiments are shown, all experiments were performed in duplicate.

TLR2-triggering was also highly effective in co-stimulating cytokine secretion in CD3-activated, freshly isolated Teff cells (Figure 3). They predominantly produced IL-2, IL-10, IL-4 and IFNγ, and cytokine production was gradually increased with increasing Pam3Cys concentrations. As expected, freshly isolated Tregs did not produce any detectable levels of these cytokines (data not shown). Also the *in vitro *expanded Tregs did not produce any cytokines while the *in vitro *cultured Teff cells (Figure 3) already produced high amounts of cytokines in the absence of Pam3Cys (*i.e. *only in the presence of anti-CD3). Adding increasing amounts of Pam3Cys to these Teff cells did not significantly affect cytokine production.

**Figure 4:**
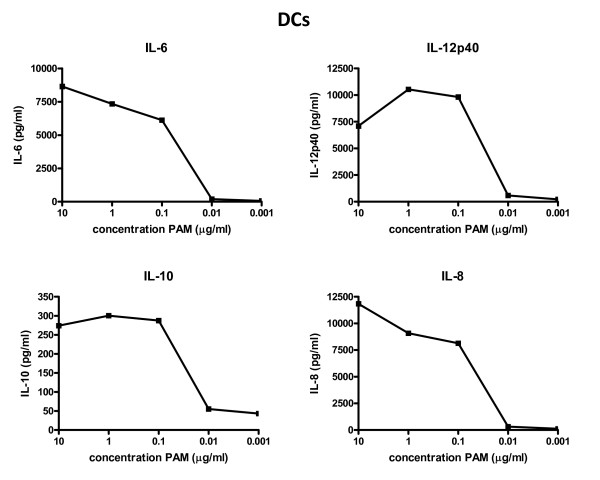
**Cytokine production of DCs upon TLR2-triggering**. Production of IL-6, IL-12, IL-10 and IL-8 by DCs after stimulation with Pam3Cys. All cytokine profiles were determined by Luminex analyses. Representative results of 2 independent experiments are shown, all experiments were performed in duplicate.

Analysis of Pam3Cys-stimulated DCs indicated that they readily produce multiple cytokines, including high amounts of IL-6 and IL-12p40 (Figure 4). Maximal cytokine production by DCs already reaches a plateau at relatively low amounts of Pam3Cys (0.1 μg/ml). Overall, the TLR2-induced cytokine profiles of the freshly isolated Teff cells and the DCs nicely correlate with a cytokine profile needed for the induction of an immune response [19-22]. Collectively, these results show that Tregs, DCs as well as Teffs can express functional TLR2. DCs are most capable of sensing low amounts of TLR2 ligand. In agreement with the current understanding that activated Teff cells are less dependent of (co-)stimulation, we show that they are less sensitive to TLR2 co-stimulation than naive Teff. In contrast, Treg remain relatively unresponsive to TCR stimulation and rely on co-stimulation in order to enter a proliferative state.

### Effect of TLR2 triggering on Treg function

**Figure 5:**
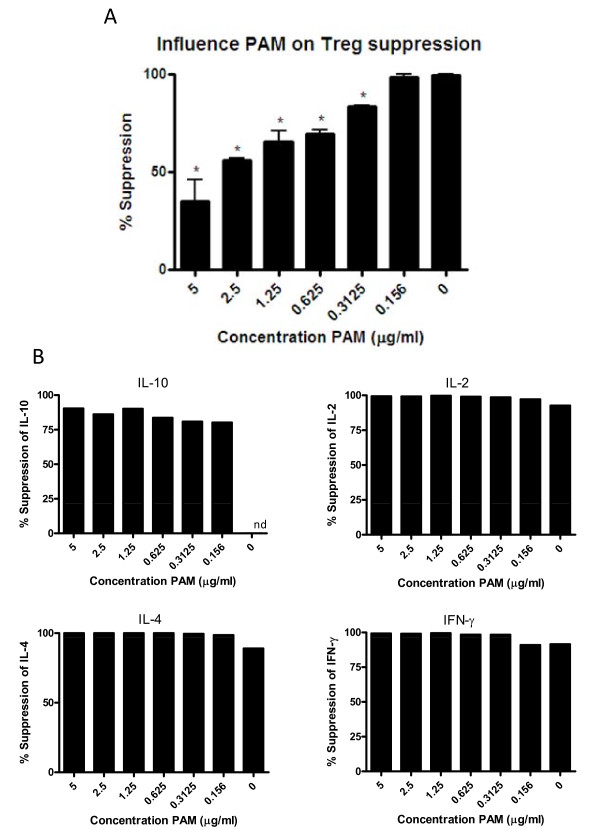
**TLR2 triggering effects Treg suppression**. (A) Suppression assays were performed with wild type Treg and TLR2-deficient Teff in the absence or presence of Pam3Cys, in which the read-out is Teff cell proliferation. Pam3Cys inhibited Treg-mediated suppression in a dose-dependent manner. (B) Analysis of the effect of Pam3Cys on IL-2, IL-4, IL-10 and IFNγ cytokine production. Increasing concentrations of Pam3Cys did not restore cytokine production (nd, not detectable). Data representative of 2 experiments are shown. The error bars represent standard deviations of duplicate measurements.

Recently, we as well as others reported that triggering of TLR2 on murine Tregs with a relatively high dose of Pam3Cys in combination with TCR signaling induced significant proliferation in the otherwise non-proliferative Treg subset [14,18]. Strikingly, Treg proliferation was accompanied by a temporal loss of the ability of the Tregs to suppress the proliferation of the Teff cells. To analyze the effects of TLR2 on Treg function in more detail, we titrated increasing concentrations of the TLR2-ligand Pam3Cys in a suppression assay and determined its impact on Teff cell proliferation and on cytokine production, using CFSE-labeled TLR2-deficient Teff cells co-cultured with wildtype Tregs. In contrast to 3H-tritium-Thymidine incorporation assay, the use of CFSE-labeled Teff cells allows to distinguish between Treg and Teff proliferation. As shown in Figure 5A (representative raw data are shown in Additional file 1), very low TLR2-ligand concentrations (<0.2 μg/ml) hardly affected the Treg mediated suppression of Teff proliferation. In line with our previous results, a higher dose of TLR2-ligands (>2 μg/ml) induced a clear loss of Treg suppressive activity as reflected by increased Teff proliferation. The addition of Tregs to the Teff cell cultures blocked proliferation as well as cytokine production. In contrast to its effect on proliferative capacity, the addition of Pam3Cys to these cultures failed to restore cytokine production by Teffs (Figure 5B). These results indicate that with respect to Treg mediated suppression of Teff cells, suppression of proliferation and suppression of cytokine production should be regarded as two separate events.

### Effect of TLR2 triggering on immune cell subsets on Treg mediated immune suppression

To determine the contribution of TLR2 triggering on the different cell types present in a suppression assay (Tregs, Teffs and APCs), we performed suppression assays combining cells isolated from TLR2-deficient mice and wildtype mice. This setup ensures that any of the observed effect of the TLR2-ligands is mediated through TLR2 triggering on the cells purified from the wildtype mice. We focused on Treg-mediated suppression of Teff proliferation as the read out system.

**Figure 6:**
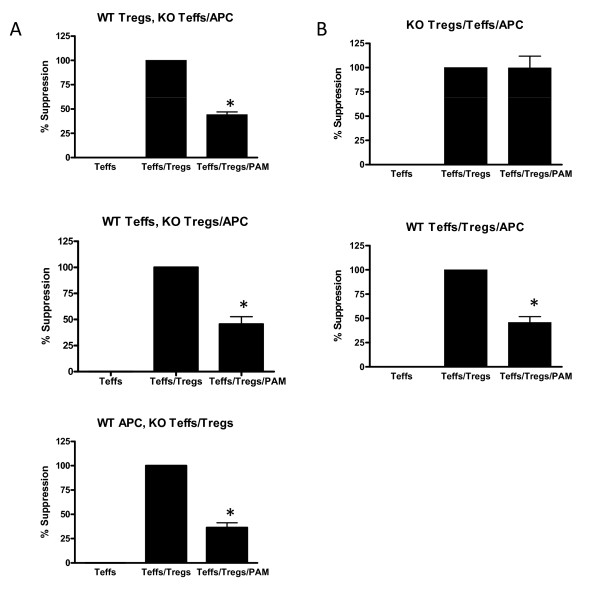
**Treg suppression assay using wildtype and TLR-2 knockout cell types**. (A) Suppression assays were performed with different combinations of wildtype and TLR2-deficient Treg, Teff or APCs. In each experiment only one of the three cell types was derived from wild type mice and thus able to respond to Pam3Cys. Data show that Pam3Cys released Treg-mediated suppression in every condition and hence affected all cell types directly. (B) Suppression assays with all cells being either TLR2 knockout or wildtype show maximal suppression and maximal release from Treg mediated suppression, respectively. Representative results of 2 experiments are shown. The error bars represent standard deviations of duplicate measurements.

TLR2 triggering on wildtype Tregs in the presence of TLR2-deficient Teff and APC resulted in a 57% reduction in the suppression of Teff cell proliferation (Figure 6A). Similarly, TLR2-signaling on Teffs (TLR2-deficient Tregs and DCs) or DC (TLR2-deficient Teff and Treg) inhibited Treg-mediated suppression to 55% and 65% respectively. A similar level of reduction in suppression was also observed when all cells were derived from wildtype origin. Control suppression assays performed with only TLR2-deficient cells demonstrated the specificity of the TLR2-effect (Figure 6B). These data thus demonstrate that TLR2 is important in controlling activation of immune cells and that TLR2 acts on each of the three cell types present in the suppression assay; Tregs, Teffs and APCs.

### Effect of Legionella pneumophila on immune cell proliferation, cytokine production and Treg suppression

**Figure 7:**
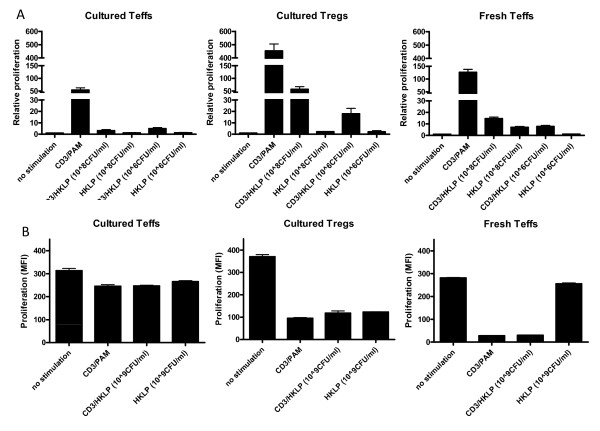
**Effect of HKLP titration on proliferation of fresh versus cultured Teff cells and cultured Tregs**. Cells were stimulated with different concentrations of HKLP in the absence or presence of anti-CD3. After 4 days proliferation was determined by analyses of thymidine incorporation (A) or CFSE dilution, displayed as MFI values (B). Cultured Treg proliferated in response to HKLP in a dose-dependent manner. Freshly isolated Teff also slightly responded to HKLP, while no effects were observed in cultured Teff. Representative results of 3 independent experiments are shown. The error bars represent standard deviations of duplicate measurements.

**Figure 8:**
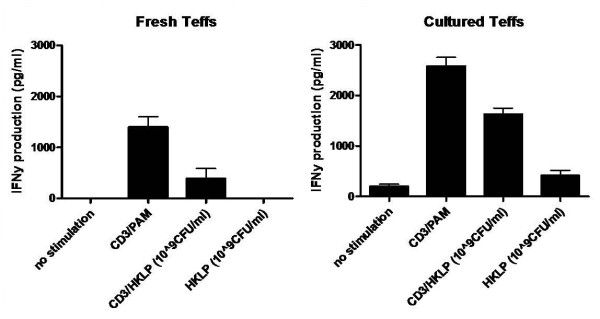
**IFNγ cytokine production by HKLP stimulation**. Cultured Teff cells and freshly isolated Teff cells were stimulated with 10^9 CFU/ml HKLP in the absence or presence of anti-CD3. Representative results of 2 independent experiments are shown. The error bars represent standard deviations of duplicate measurements.

So far, the experiments were performed with the synthetic TLR2 ligand Pam3Cys. We repeated these experiments with the natural pathogen *Legionella pneumophila*, which is known to contain high amounts of TLR2 ligands [23]. As shown in Figure 7, the proliferative capacity of the cultured Teff cells and Tregs were similar to the data found with Pam3Cys. The cultured Teff cells were hardly responsive to the HKLP, and this did not change upon increasing pathogen concentration. The naive CD4+ Teff cells were highly responsive to HKLP in a dose-dependent manner. Both freshly-isolated and cultured Teff produced IFN-γ upon HKLP stimulation (Figure 8).

**Figure 9:**
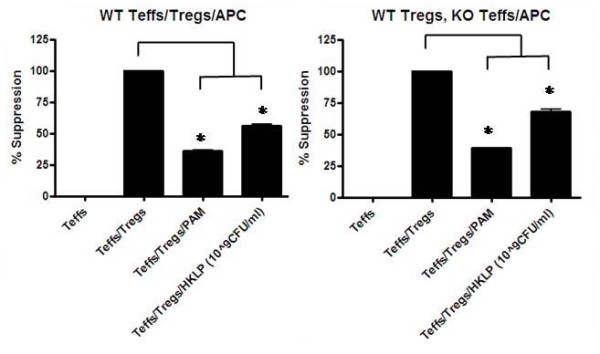
**Treg suppression with HKLP stimulation**. Suppression assays were performed with two combinations of wildtype and TLR2-deficient cells. When all cells were from wildtype mice both Pam3Cys and HKLP inhibited Treg-mediated suppression (left panel). Similar results were obtained when TLR2-deficient Teff and APC were cultured in the presence of wildtype Treg, indicating that HKLP acted on the Treg themselves. Representative results of 2 experiments are shown. The error bars represent standard deviations of duplicate measurements.

To further investigate the effect of HKLP on Treg, suppression assays were performed. The results shown in Figure 9 indicate that HKLP abrogated the Treg-mediated suppressive capacity even in conditions where Tregs are the only TLR2 expressing cells. These data imply that the immune modulatory effect of Pam3Cys can be mimicked by a natural pathogen containing high amounts of TLR2 ligand. It is likely that during normal infections of *Legionella pneumophila *the suppressive capacity of the Tregs can at least in part be abrogated by directly acting on the Tregs themselves.

## Discussion

The complexity of signals received and produced by our immune system has puzzled immunologist for many years. The re-discovery of the suppressor T cells mostly indicated as Tregs has increased our comprehension of immunity in health and disease. In immune homeostasis, immune activation events are kept in balance by many regulatory mechanisms like suppressors of cytokine signaling (SOCS), CTLA-4, suppressive cytokines like IL-10 and TGF-β, and importantly Tregs. However during infections, strong immune activation signals are released, including a set of pathogen-associated molecular patterns. These pathogen derived determinants are able to induce the activation of amongst others TLRs, one of the main families of receptors involved in pattern recognition. This will result in a strong inflammatory immune response capable of dealing with the pathogenic challenge. More recently, TLR agonists have been shown to also act directly on Treg and 'lift' the suppressive pressure on other immune cells. We demonstrated that TLR2 triggering on Tregs in combination with TCR signaling induces proliferation of the Tregs and reduces their suppressive capacity [14]. Similar data were later reported by Liu *et al. *[18] . Recently Oberg *et al. *[24], examined the influence of different TLR2 ligands on human Tregs. They show similar results for 75% of the used human donors, namely direct abrogation of the Treg suppressive capacity. They also observed individual differences in efficacy between different TLR2 ligands. Above all, Zhang *et al. *[25] currently tested the abrogation of Treg-mediated suppression in an *in vivo *tumor-bearing mouse model. They report that injection of the mice in combination with pretreatment of the Treg cells with Pam3Cys, induces a marked reduction of Treg cells in the lymph nodes and a diminished suppressive activity of the remaining Tregs present in these mice. On the contrary, another researcher group has published that TLR2 does not abrogate the Treg suppressive function [26]. The different findings might be explained by the difference in model systems, cell sources, experimental setups and as described by Oberg *et al. *[24] the expression pattern of TLR1/TLR2.

The loss of suppression by TLR2 triggering on Treg facilitates the induction of adaptive immune responses. At the same time, TLR2-stimulated Tregs expand and have been suggested to limit excessive tissue pathology and to prevent potential autoimmune events once the pathogen is efficiently cleared. To further assess the role of TLR2, we investigated the effects of TLR2 stimulation on Treg and other immune cells known to be involved in the adaptive immune response; Teff cells and the professional antigen presenting DCs. Freshly isolated splenic DCs express TLR2, but the freshly isolated Tregs and Teff cells only express TLR2 upon activation. Interestingly, all freshly isolated cells respond to the TLR2-ligand Pam3Cys, however after *in vitro *culturing, only the Tregs remained responsive to TLR2-stimulation in lower concentrations. TLR2 on naive Teff cells enhanced Teff cell proliferation indicating that TLR2 acts as a co-stimulatory signal, similar to CD28. Cultured Teff cells did not seem to respond to TLR2 triggering. These data are in line with the idea that primed Teff cells are less dependent on co-stimulation by TLR2, similar to co-stimulation by CD28, though the anergic Treg subset requires more than TCR triggering alone in order to enter a state of proliferation.

When analyzing the effects of TLR2-stimulation of Tregs in more detail, we observed a difference between suppression of proliferation and suppression of cytokine production by the Treg. Interestingly, TLR2-treated Treg still suppressed cytokine secretion, but lost their ability to suppress the proliferation of Teff cells. Apparently, the process of suppression of cytokine secretion is different from that of suppression of proliferation. This is in line with data of Shevach *et al. *showing that mature DCs abrogated Treg-mediated suppression of Teff cell proliferation, while cytokine secretion was still suppressed [10]. Moreover, DCs stimulated the proliferation of Treg, similar as observed after TLR2 stimulation. It remains intriguing why such highly different signals (TLR2 versus mature DCs) would yield similar phenotypes in the Tregs. One possibility would be that the Tregs require a certain threshold of co-stimulatory signals. Indeed, it has been shown that TLR2 can substitute for CD28 with respect to co-stimulatory signals required for T cell stimulation [21]. Once the combination of co-stimulatory signals and TCR triggering is sufficient, the Treg will suppress only cytokine secretion by Teff and will start to proliferate themselves. Why would Treg allow Teff proliferation whilst inhibiting Teff cytokine production? Possibly cytokine secretion is a far more dangerous effector mechanism as compared to Teff proliferation.

Since TLR2 effected suppression of proliferation, we addressed the effects of TLR2 on each of the cellular subsets present during the suppression assay. Our results show that TLR2 can abrogate the suppression of proliferation by acting on each subset being Treg, Teff or DC. TLR2 triggering on Treg induces their proliferation and temporal inhibition of their suppressive capacity, while on Teff cells TLR2 has a co-stimulatory role. TLR2 triggering on DCs results in the production of cytokines like IL-6 that have been shown to release Teff from Treg mediated suppression [27]. We examined IL-6 production in our co-culture experiments combining Teffs, Tregs and DCs for wild type and TLR2-knock out origin (data not shown). Although the presence of IL-6 coincides with the abrogation of Treg-mediated suppression of Teff proliferation in the wild type conditions, Teffs also start to proliferate when Teffs/DCs are TLR2-deficient. We therefore conclude that IL-6 is able to stimulate the proliferation of Teffs but is not absolutely required for abrogation of Treg-mediated suppression of Teff proliferation. By acting on all multiple immune cell subsets involved in adaptive immunity, TLR2 stimulation launches an effective immune response. A potent increase of regulatory mechanisms will have to occur to suppress any potential autoimmune events. The observed expansion of Tregs following immune activation shown here and in our previous work represents one of these feed-back mechanisms. The increased expansion of Treg may prevent immune pathology and help to dampen the immune response once the cause of the immune challenge has been eliminated.

The question arose whether the effect of synthetic TLR2 ligand could also be observed during natural infections. The gram-negative micro-organism *Legionella pneumophila *is the causing agent of a severe pneumonia. Immune recognition of this microorganism has been reported to be dependent on TLR2 signaling and not on TLR4 signaling [15,16]. Therefore we repeated the experiments with heat-killed *Legionella pneumophila *(HKLP) to confirm our Pam3Cys findings. Indeed the effect of TLR2 triggering on all different immune cells could be confirmed when using HKLP in our assays. Most importantly the suppressive capacity of the Tregs could be abrogated in the presence of HKLP. These data indicate that TLR2 triggering potently modulates immune function during bacterial infections. However, it should be considered that besides effects on TLR2, stimulation of other pathogen recognition receptors could drastically influence the outcome of induced immune responses against specific pathogens. Recognition of *Legionella pneumophila *is mainly relying on TLR2 triggering. Other pathogens likely trigger other receptors that could possibly effect Treg suppression.

## Conclusions

Our findings indicate that Treg-mediated suppression of T helper cell proliferation is functionally distinct from suppression of cytokine secretion. TLR2 acts on multiple cells of the adaptive and innate immune system thereby providing direct immune stimulation of Teffs and DCs while simultaneously inhibiting the suppressive abilities of Tregs. These effects are not only observed for a synthetic TLR2 ligand, but could be confirmed with the natural TLR2 ligand containing bacteria *Legionella pneumophilia*. Further understanding of the precise mechanisms involved, will most likely contribute to new strategies for immune-based intervention strategies.

## Methods

### Mice

The wildtype C57BL/6 mice were obtained from Charles River WIGA (Sulzfeld, Germany) Gmbh. TLR2 knockout on C57BL/6 background mice were kindly obtained from S. Akira (Osaka University, Osaka, Japan). All animal experiments were approved by the Animal Experimental Committee of Radboud University Nijmegen Medical Centre and were performed in accordance with institutional and (inter)national guidelines.

### Antibodies and flow cytometry

Directly labeled monoclonal antibodies used for staining by anti-CD4-APC or -FITC (clone L3T4), anti-CD25-FITC or -PE (clone 7D4), anti-CD11c-FITC (HL3), anti-Streptavidin-PerCp, and their isotype controls were obtained from BD Biosciences - Pharmingen. Anti-mTLR2-biotine (clone T2.5), anti-mFoxP3-biotine (clone FJK-16S) and the isotype control were obtained from eBioscience. Analysis of cell surface markers on lymphocytes was performed using FACScalibur (BD) and CELLQuest software (version 3.3; BD Biosciences - Pharmingen).

### T cell purification and analysis

Spleens from wildtype or TLR2 knockout mice were mashed and filtered, and CD4+ T cells were purified using anti-mouse-CD4 Microbeads (MACS, Miltenyi Biotec), resulting in a enriched CD3+CD4+ T cell population. Naive CD4+CD25low Teff cells and naive CD4+CD25high Treg cell subsets were obtained by flow cytometry purification of the pre-sorted CD4+ T cells; CD4 cells were stained with FITC-conjugated CD4 mAb (BD bioscience, clone L3T4) and PE-conjugated CD25 (BD biosciences, clone 7D4). Cell sorting was performed on a Coulter Altra HyPerSort cell sorter. Both naive CD4+CD25low T cells and naive CD4+CD25high T cells were 98% pure, based on CD25 expression pattern. Determining the purity by using biotinilated-FoxP3 Ab (eBiosciences, clone FJK-16S) and streptavidine-PerCp Ab (BD bioscience) showed that from the CD25high FACSsorted cells, 67% of the cells were FoxP3+, therefore Tregs. Sorted cells were used directly in several assays or kept in culture as described below. After several weeks of culture the purity of the Treg cell line as well as the Teff cell line (referred in this paper to the cultured Tregs or cultured Teff cells) was 96% or higher (see Additional file 2).

### Treg and Teff cell culture and suppression assay

FACS-sorted purified CD4+CD25+ T cells and CD4+CD25-T cells are kept in culture for several weeks. Each cell-line is cultured in 10^4 cells per well of a 96-well plate, and were stimulated weekly with 5*10^4 irradiated CD4-MACS bead depleted splenocytes per well, in 2 μg/ml Pam3Cys (EMC microcollections, Germany), 1 μg/ml anti-CD3 (145-2C11; BD Biosciences - Pharmingen), and 120 IU (international units) IL-2/ml complete medium. The cells were washed 3 days after each stimulation and maintained in culture medium supplemented with 120 IU IL-2/ml. When necessary, dead cells were removed by ficoll density gradient. Cultured Tregs or Teff cells were used in assays at least 6 days after stimulation, in this case the Tregs and Teff cells are in a resting state when used in all assays.

Suppression assays were performed as follows; freshly sorted (wildtype or TLR2 knockout) CD4+CD25-naive T cells (20*10^3 per well) and either cultured or freshly isolated (wildtype or TLR2 knockout) Tregs (20*10^3 per well) were mixed and cocultured for 3 days with 20*10^3 irradiated (wildtype or TLR2 knockout) APCs per well. If indicated, the T cells were stimulated with TLR ligand Pam3Cys (5 μg/ml, EMC microcollections GmbH) or HKLP (10^6-10^9 CFU/ml of heat-killed *Legionella pneumophila*; InvivoGen) with or without soluble anti-CD3 (1 μg/ml, 145-2C11; BD Biosciences - Pharmingen) in complete medium. After 3 days of coculture, supernatant was collected for cytokine analysis. In addition, the suppression of proliferation was monitored by analyzing the CFSE-labeled (1 μM) freshly sorted (wildtype or TLR2 knockout) CD4+CD25-naive T cells. CFSE fluorescence intensity was measured by flow cytometry.

### Calculation of suppression

The amount of suppression is calculated by the amount of proliferation or cytokine production. In each assay we include 5 conditions (see Additional file 3) by which we calculate the suppression, using the mean fluorescent intensity of the CFSE-labeled Teff cells. The first condition (background) is a coculture of CFSE-labeled Teff cells, APCs and anti-CD3 stimulation (1 μg/ml). This condition indicates the level of Teff cell proliferation induced by anti-CD3 in the presence of APC. The second condition (0% suppression) is a coculture of CFSE-labeled Teff cells, APCs, anti-CD3 stimulation (1 μg/ml) and Pam3Cys (concentration used between 10 to 0.01 μg/ml). This condition represents maximum of Teff cell proliferation. The next condition consists of a coculture of CFSE-labeled Teff cells, APCs, Tregs and anti-CD3 stimulation (1 μg/ml), which is used to achieve maximal suppression (set to 100% as a reference). The last condition consists of a coculture of CFSE-labeled Teff cells, APCs, Tregs, anti-CD3 stimulation (1 μg/ml) and Pam3Cys (concentration used between 10 to 0.01 μg/ml), indicated as 'x% suppression'. This x% suppression is calculated by the following formula; ((MFI x% - MFI 0%)*100)/(MFI max - MFI 0%). For all data presented the difference between the 0% suppression and max suppression was at least 150 in MFI. In the case of cytokine production, we used the same formula with amount of cytokines produced as principle parameter.

### Cytokine measurements

For the detection of cytokines in culture supernatant we used the Mouse Th1/Th2 Bio-Plex Cytokine Assay (#171-F11081, Bio-Rad, Hercules, CA). All procedures were performed according to the manufacturer's instructions.

### Proliferation assay

Freshly FACS-sorted or cultured CD4+CD25+Treg and CD4+Tconv cells were cultured for 4 days with a range of Pam3Cys concentrations (between 0 μg/ml-5 μg/ml) or HKLP (10^6-10^9CFU/ml of heat-killed *Legionella pneumophila*; InvivoGen) with or without soluble CD3 stimulation (1 μg/ml). After 4 days the proliferation was measured by overnight (20 hours) thymidine incorporation.

### Statistical analysis

The data are analysed using a one-way ANOVA test, to test the differences between groups (PRISM software version 4.0; GraphPad, San Diego, CA, USA). Statistical significance is inferred at P < 0.05. Significant differences are indicated with an asterisks (*).

## List of abbreviations

**APC: **antigen presenting cell; **DC: **dendritic cell; **FoxP3: **fork-head box protein 3; **HKLP: **heat-killed *Legionella pneumophila*; **HSP60: **heat-shock protein 60 **IFNγ: **interferon gamma; **IL-...: **interleukin...; **MFI: **mean fluorescent intensity; **NLR: **NOD like receptor; **Pam3Cys: **Pam3CysSK4; **PAMPs: **pathogen associated molecular patterns; **RA: **rheumatoid arthritis; **RLR: **RIG-I like receptor; **Teff(s): **effector T cell(s); **TGFβ: **transforming growth factor beta; **TLR...: **toll like receptor...; **Treg(s): **regulatory T cell(s).

## Authors' contributions

WWCvM contributed to this article by performing most of the experiments. LWT and JMB contributed by mostly doing the cytokine assays, and LWT also performed several suppression assays. SN contributed by giving intellectual input. RPMS and GJA are both equally responsible for the total study concept, study design and guidance. GJA is the daily supervisor and the Principle Investigator. All authors have read and approved the final manuscript.

## Supplementary Material

Additional file 1 **Suppression assay with increasing Pam3Cys concentrations**. Suppression is determined by CFSE dilution of the naive Teff cells. These data show the CFSE peaks obtained after 3-4 days culture with or without Pam3Cys (5 μg/ml). Loss of Treg-mediated suppression resulted in more Teff proliferation, indicated by a peak shift to the left. Click here for file

Additional file 2 **Purity of isolated Tregs and Teffs**. The Teff cells and Tregs were MACS-sorted from total splenocytes based on CD4 expression and thereafter FACS-sorted into CD25 high or low expressing cells. After several weeks in culture, the purity of Teff and Treg was 98% and 96% respectively. Click here for file

Additional file 3 **Conditions used in suppression assay**. This list shows all conditions always used in suppression assays, and which cells/stimulations are in each condition. Click here for file
