# Supplementary figures and images for: Multifaceted effects of synthetic TLR2 ligand and Legionella pneumophilia on Treg-mediated suppression of T cell activation
Source: BMC Immunol. 2011 Mar 24;12:23. doi: 10.1186/1471-2172-12-23 (PMC3078900; doi:10.1186/1471-2172-12-23)

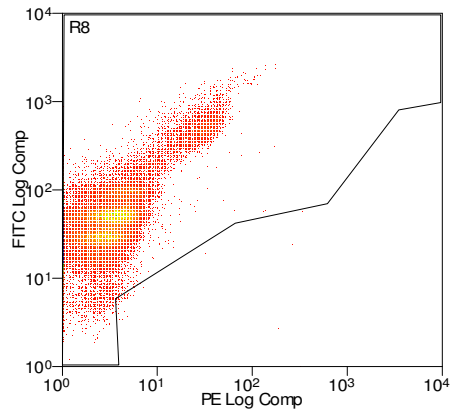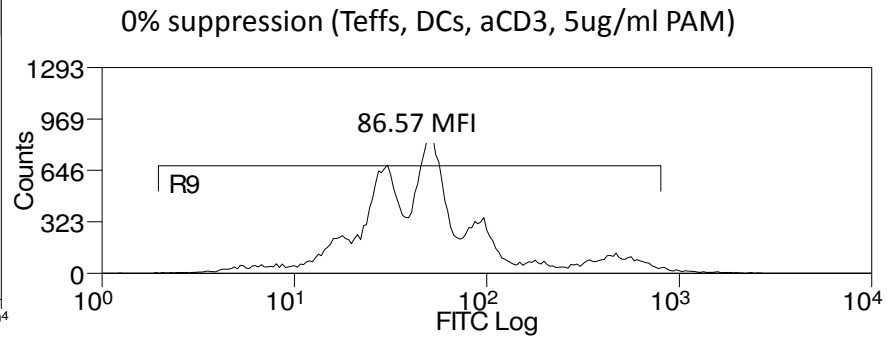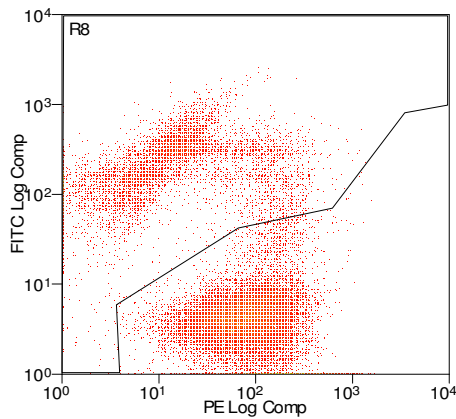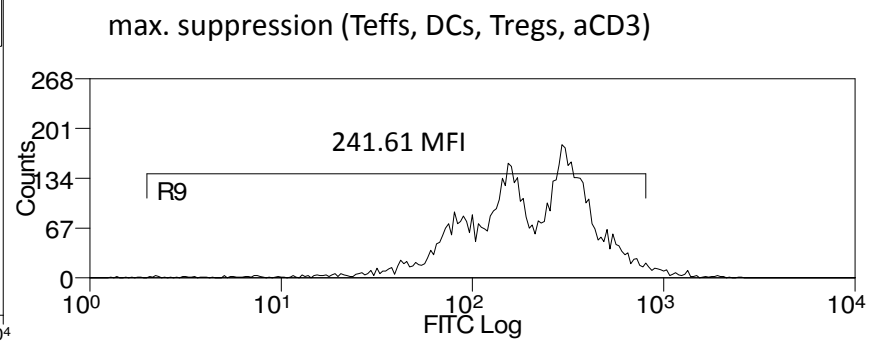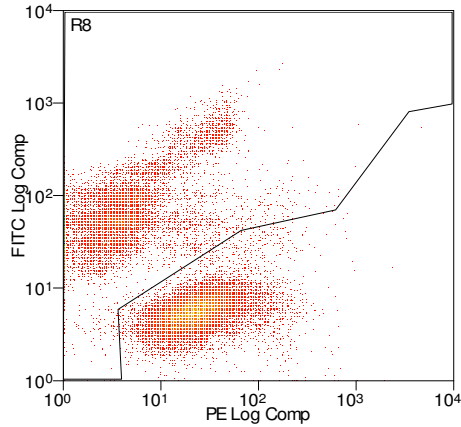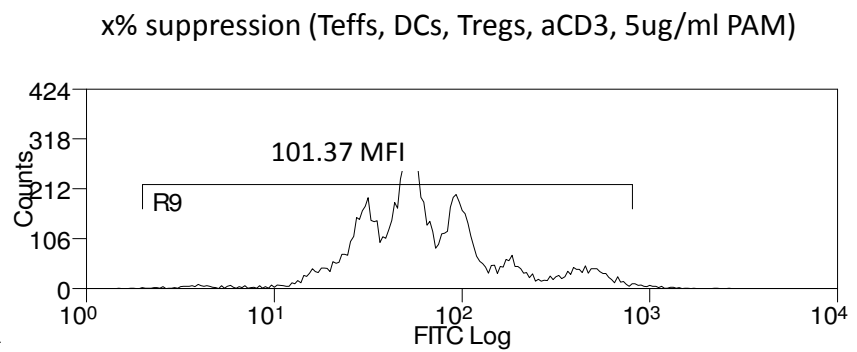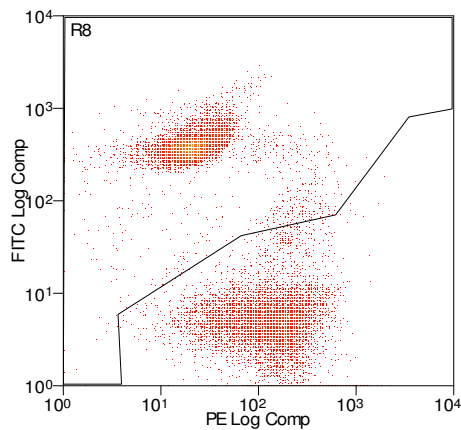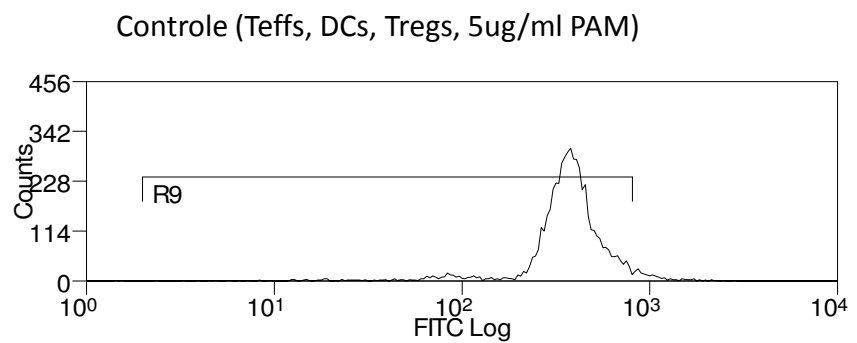

Supplement: Additional file 1 — Suppression assay with increasing Pam3Cys concentrations. Suppression is determined by CFSE dilution of the naive Teff cells. These data show the CFSE peaks obtained after 3-4 days culture with or without Pam3Cys (5 μg/ml). Loss of Treg-mediated suppression resulted in more Teff proliferation, indicated by a peak shift to the left. [file 1471-2172-12-23-S1.PDF]

Cultured Teff cell-line

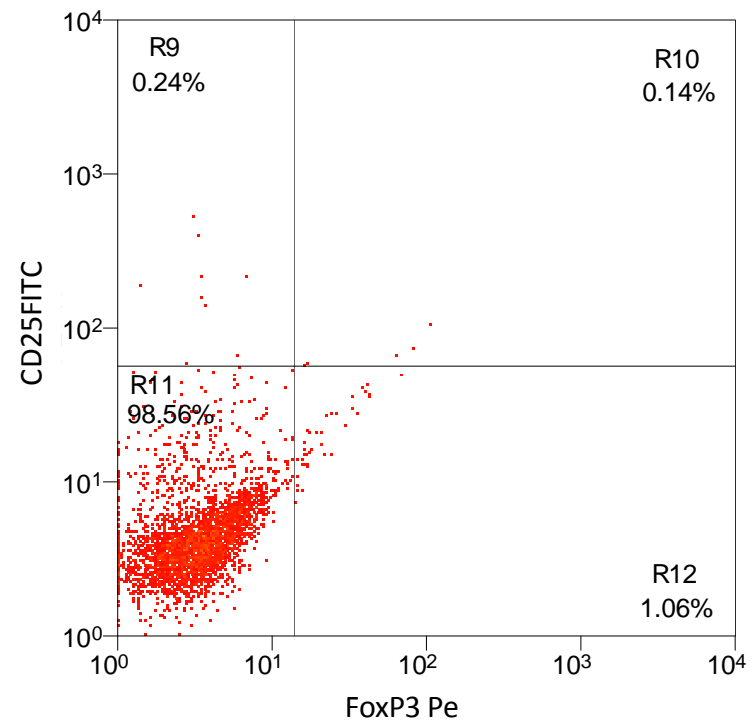

Cultured Treg cell-line

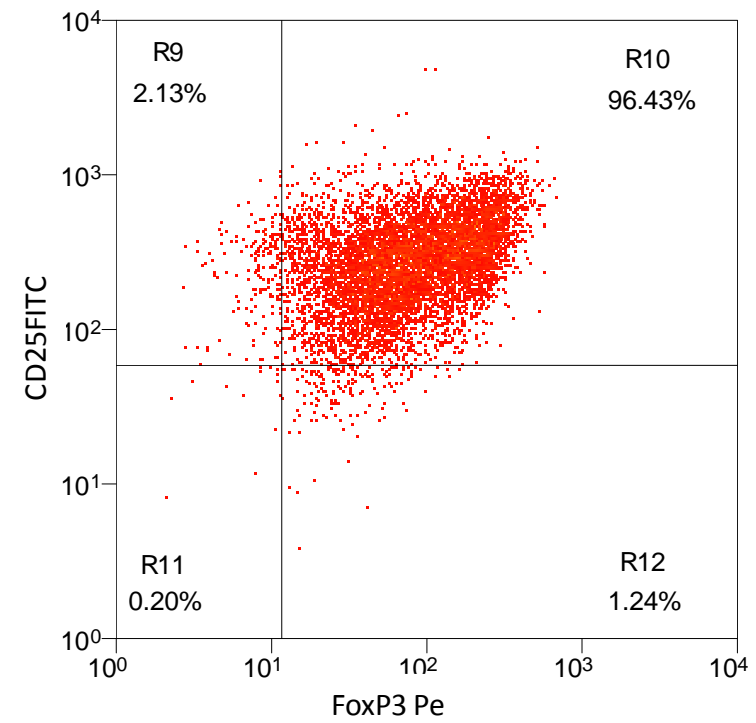

Supplement: Additional file 2 — Purity of isolated Tregs and Teffs. The Teff cells and Tregs were MACS-sorted from total splenocytes based on CD4 expression and thereafter FACS-sorted into CD25 high or low expressing cells. After several weeks in culture, the purity of Teff and Treg was 98% and 96% respectively. [file 1471-2172-12-23-S2.PDF]
