# Supplementary material for: Multifaceted effects of synthetic TLR2 ligand and Legionella pneumophilia on Treg-mediated suppression of T cell activation
Source: BMC Immunol. 2011 Mar 24;12:23. doi: 10.1186/1471-2172-12-23 (PMC3078900; doi:10.1186/1471-2172-12-23)
Supplement: Additional file 3 — Conditions used in suppression assay. This list shows all conditions always used in suppression assays, and which cells/stimulations are in each condition. [file 1471-2172-12-23-S3.PDF]

Conditions		
1	Background	Teff, APCs, αCD3
2	0% suppression	Teff, APCs, αCD3, PAM
3	max. suppression	Teff, APCs, Treg, αCD3
4	X% suppression	Teff, APCs, Treg, αCD3, PAM
5	Control	Teff, APCs, Treg, PAM
